# Do sexual minorities believe that they die earlier? Results from a large, representative survey

**DOI:** 10.1186/s12877-023-04453-5

**Published:** 2023-11-14

**Authors:** André Hajek, Elzbieta Buczak-Stec, Hans-Helmut König

**Affiliations:** Department of Health Economics and Health Services Research, Hamburg Center for Health Economics, University Medical Center Hamburg-Eppendorf, Hamburg, Germany

**Keywords:** LGBT, Sexual orientation, Perceived longevity, Subjective life expectancy, Sexual minorities

## Abstract

**Background:**

While various consequences of belonging to sexual minorities have been examined – it remains completely unclear whether sexual minorities believe that they die earlier. Thus, our aim was to investigate the association between sexual orientation and expected longevity.

**Methods:**

Data from the German Ageing Survey, a nationally representative sample, were used (year 2014, n = 6,424 individuals; mean age: 63.6 years). It included individuals residing in private households aged 40 years and over in Germany. Sexual orientation (heterosexual; sexual minorities including homosexual, bisexual, or other) served as key independent variable. As outcome, we used the expected life expectancy. In multiple linear regressions it was adjusted for gender, age, education, marital status, labour force participation, BMI, smoking status, alcohol intake, sports activities, physical functioning, self-rated health and the number of chronic conditions.

**Results:**

Adjusting for sociodemographic, lifestyle-related and health-related factors, our study showed that sexual minorities reported a lower expected longevity (β=-0.69, p = .02) compared to heterosexuals. This association remained nearly the same in robustness checks.

**Conclusion:**

After adjusting for various other factors, our findings showed a lower life expectancy among sexual minorities compared to heterosexuals. Efforts are required to make sexual minorities believe in a high life expectancy (e.g., increased optimism or reduced perceived discrimination) – which in turn can help to increase their actual longevity and successful ageing. Future research is required to explore underlying mechanisms (such as expected stigma in later life).

**Supplementary Information:**

The online version contains supplementary material available at 10.1186/s12877-023-04453-5.

## Background

About 7% of community-dwelling middle-aged and older individuals living in Germany self-identified as sexual minorities (lesbian, gay, bisexual, transgender and other) [1]. Nevertheless, there is still limited knowledge regarding individuals belonging to sexual minorities. According to a study conducted by Cochran et al. [2], such individuals (in middle age) have a higher likelihood of mental disorders compared to heterosexuals - possibly for reasons of stigmatization. They also have a higher likelihood of not having a partner in later life [3]. Additionally, such individuals may have had adverse childhood experiences (e.g., experiences of social exclusion or discrimination). Following the stress theory, such enduring stress can contribute to long-lasting negative effects on their overall well-being or health [4]. The stress of sexual minorities can be the result of experiences of prejudices, personal rejection, discrimination or stigma [4, 5]. Grounded on the minority stress theory (which refers to chronic exposure to this excessive stress), several studies have examined the psychosocial consequences. For example, a recent study [6] showed that sexual minorities reported higher loneliness scores compared to heterosexuals among community-dwelling individuals aged 40 years and over in Germany. Older sexual minorities can also experience discrimination in the healthcare system [7], particularly in nursing homes [4, 8, 9]. Former research also showed that structural stigma is linked to mortality amongst individuals reporting past-year same-sex sexual partners [10]. Another study [11] found a greater all-cause mortality in sexual minorities compared to heterosexuals after adjusting for demographic covariates based on NHANES data. Such effects mainly disappeared after adjusting for lifestyle and health-related factors [11].

While various consequences of belonging to sexual minorities have been examined – as outlined above, it remains unclear whether sexual minorities believe that they die earlier. Thus, our aim was to investigate the association between sexual orientation and expected longevity based on nationally representative data. In accordance with the minority stress theory, we assume that sexual minorities believe that they die earlier (compared to heterosexuals). For example, the higher loneliness reported by middle-aged and older sexual minorities [6] could contribute to lower perceived longevity – as previously shown among individuals in later life [12].

Knowledge regarding a potential association between sexual orientation and perceived life expectancy is important because it could become a self-fulfilling prophecy [12]: Individuals who may think that they die early may subsequently have bad lifestyle habits (such as smoking, sedentary behavior or alcohol intake) [13, 14]. Such bad lifestyle habits may result in chronic conditions. Additionally, the perceived nearness to death may contribute to distress – which could also increase the number of chronic conditions [12]. Ultimately, our results may highlight the need for changing attitudes among sexual minorities (e.g., increase optimism) and may also highlight the importance of reducing stressors (such as discrimination or perceived stigma) in this group.

## Methods

### Sample

We used data from the German Ageing Survey (DEAS) wave 5 in 2014. The DEAS survey is a nationally representative sample of individuals living in Germany aged 40 and up, also known as the “second half of life”. The individuals were chosen using a national probability sampling procedure. The main inclusion criteria are that the individuals be at least 40 years old and live in a private household. As a result, people living in institutions were not included. The DEAS study, which began in 1996, is designed in a cohort-sequential fashion. It covers a wide range of topics, including, among other things, the transition to retirement, satisfaction in old age, general well-being, and health. Health issues and refusal were the most common reasons for leaving the DEAS study in upcoming waves. The response rate in wave 5 was 25% for first time participants and 61% for individuals who already participated in former waves.

Following an interview, which include, for example, sociodemographic variables, 8,039 individuals correctly completed a questionnaire in the fifth wave. A complete deletion of the drop-off information (15 cases) was only carried out if the person who filled out the drop-off was most probably not the target person with whom the personal interview was conducted beforehand.

Moreover, 649 individuals (8.1%) did not answer the question regarding the expected longevity and 26 cases (0.3%) were deleted in data preparation (consistency checks that took place, e.g., when the expected longevity was lower than the actual age) by the DEAS staff. We further removed eight cases because the expected longevity was extremely high (200 years; one case each: 400 years, 555 years, 646 years, 999 years) and removed one case where the expected life expectancy was lower than the chronological age. Furthermore, 807 individuals (10.0%) did not respond to the question regarding sexual orientation and 15 cases (0.2%) were removed in data preparation by the DEAS staff. These latter 15 cases are those where the statements in the paper questionnaire could not be clearly assigned to an answer category (i.e. these participants placed their crosses in such a way that no clear assignment to one of the four categories was possible). Since missing values also occurred in the other independent variables, the final analytical sample equaled 6,424 individuals. Moreover, further details with respect to the DEAS study are provided by Klaus et al. [15].

### Dependent variable

As in other large cohort studies, individuals were asked to estimate their expected life expectancy. More specifically, the question was phrased as follows: ‘What age do you think you will live to?’ [_ _ _ years].

### Key independent variable

Comparable to other survey studies [16], participants were asked “How would you describe your sexual orientation?“ There are four possible responses: heterosexual, homosexual, bisexual, or other. In line with former research (e.g., [6, 16]), the sexual orientation was divided into two categories: heterosexual and sexual minorities (gay/lesbian, bisexual, and other).

### Covariates

Grounded on former studies (e.g., [17, 18]) and also based on theoretical considerations, sociodemographic, lifestyle-related and health-related covariates were included in our regression model.

With regard to sociodemographic factors, we included: gender (men; women), age (in years), educational level (ISCED-classification: low (0–2), medium (3–4), and high (5–6) education [19]), marital status (single; divorced; widowed; married, living separated from spouse; married, living together with spouse), and labor force participation (employed; retired; other: not employed).

With regard to lifestyle-related factors, we included: body-mass-index (BMI; based on self-reported weight and height), smoking status (yes, daily; yes, sometimes; no, not anymore; never smoking), alcohol intake (daily; several times a week; once a week; 1–3 times a month; less often; never), and the frequency of sports activities (daily; several times a week; once a week; 1–3 times a month; less often; never).

With regard to health-related factors, we included: self-rated health (from 1 = very good to 5 = very bad), physical functioning (SF-36 subscale physical functioning [20], from 0 (worst) to 100 (best)), and a count score for physical illnesses which ranged from 0 to 11 (cardiac and circulatory disorders; bad circulation; joint, bone, spinal and back problems; respiratory problems, asthma, shortness of breath; stomach and intestinal problems; cancer; diabetes; gall bladder, liver or kidney problems; bladder problems; eye problems, vision impairment; ear problems, hearing problems). In a robustness check, it was additionally adjusted for depressive symptoms (15-item Center for Epidemiologic Studies Depression Scale, from 0 to 45, higher values reflect more depressive symptoms [21]).

### Statistical analysis

First, we calculated the sample characteristics for our analytical sample (also stratified by sexual orientation). Following that, multiple linear regressions were run to investigate the relationship between sexual orientation and perceived life expectancy, adjusting for several covariates. We also calculate effect sizes (partial eta-square) values for the key associations from regression analysis. Such values can be interpreted in the following way [22]: 0.01 as “small”, 0.06 as “medium, and 0.14 as “large”.

Given the highest verified age of about 120 years, one could question whether these are rational expectations. As a result, we removed these eight values (four cases: 150 years; one case each: 130 years, 154 years, 180 years, 190 years) in a robustness check. As part of another robustness check, the full information maximum likelihood approach (FIML) was used to deal with missing data. In a further check, all four categories (heterosexual, homosexual, bisexual, or other) of the sexual orientation variable were used – with heterosexual being the reference category.

The statistical significance level was set at p ≤ .05. The analyses were carried out using Stata 16.1 (StataCorp, College Station, TX, USA).

## Results

### Sample characteristics

Sample characteristics for the analytical sample (also stratified by sexual orientation) are given in Table 1 (sample characteristics with column percentages are shown in Supplementary File 1). In our sample, mean age was 63.6 years (SD: 11.0 years; from 40 to 95 years) and about 49.3% of the individuals were female. Additionally, 8.1% of the individuals belonged to the group of sexual minorities. Further details are given in Table 1.

**Table 1:** Sample characteristics for the analytical sample (n = 6,424 individuals; 5,903 heterosexuals, 521 sexual minorities)

Variables	Heterosexuals	Sexual minorities	Total	P-value
Expected longevity: Mean (SD)	83.9 (7.7)	83.5 (7.1)	83.9 (7.7)	0.22
Gender: N (%)				0.33
Men	2985 (91.6)	275 (8.4)	3260	
Women	2918 (92.2)	246 (7.8)	3164	
Age: Mean (SD)	63.2 (10.9)	67.6 (11.0)	63.6 (11.0)	< 0.001
Education (ISCED-97): N (%)				< 0.001
Low education (ISCED: 0–2)	267 (84.5)	49 (15.5)	316	
Medium education (ISCED: 3–4)	2940 (90.8)	297 (9.2)	3237	
High education (ISCED: 5–6)	2696 (93.9)	175 (6.1)	2871	
Marital status: N (%)				< 0.001
Married, living together with spouse	4221 (93.1)	312 (6.9)	4533	
Married, living separated from spouse	102 (95.3)	5 (4.7)	107	
Divorced	614 (91.6)	56 (8.4)	670	
Widowed	560 (87.0)	84 (13.0)	644	
Single	406 (86.4)	64 (13.6)	470	
Employment status: N (%)				< 0.001
Working	2407 (95.0)	126 (5.0)	2533	
Retired	2956 (89.1)	361 (10.9)	3317	
Other (not employed)	540 (94.1)	34 (5.9)	574	
Body-Mass-Index (BMI): Mean (SD)	26.9 (4.6)	27.3 (4.6)	26.9 (4.6)	0.06
Smoking status: N (%)				< 0.01
Yes, daily	863 (93.4)	61 (6.6)	924	
Yes, sometimes	240 (94.1)	15 (5.9)	255	
No, not anymore	2251 (92.7)	178 (7.3)	2429	
Never smoking	2549 (90.5)	267 (9.5)	2816	
Alcohol intake: N (%)				< 0.01
Daily	743 (90.0)	83 (10.0)	826	
Several times a week	1521 (93.4)	107 (6.6)	1628	
Once a week	972 (92.9)	74 (7.1)	1046	
1–3 times a month	728 (92.6)	58 (7.4)	786	
Less often	1341 (91.2)	130 (8.8)	1471	
Never	598 (89.7)	69 (10.3)	667	
Doing sport: N (%)				< 0.001
Daily	487 (92.4)	40 (7.6)	527	
Several times a week	1723 (93.8)	113 (6.2)	1836	
Once a week	1098 (93.2)	80 (6.8)	1178	
1–3 times a month	455 (92.9)	35 (7.1)	490	
Less often	711 (92.3)	59 (7.7)	770	
Never	1429 (88.0)	194 (12.0)	1623	
Physical functioning (from 0 (worst) to 100 (best): Mean (SD)	83.3 (21.8)	77.1 (25.2)	82.8 (22.1)	< 0.001
Self-rated health (from 1 = very good to 5 = very bad): Mean (SD)	2.5 (0.8)	2.7 (0.8)	2.5 (0.8)	< 0.001
Number of physical illnesses (count: 0 to 11 physical illnesses): Mean (SD)	2.5 (1.8)	3.0 (2.0)	2.5 (1.8)	< 0.001
Depressive symptoms (from 0 to 45, with higher values reflect more depressive symptoms): Mean (SD)	6.4 (5.8)	8.0 (6.6)	6.5 (5.9)	< 0.001

*Notes*: P-values are based on χ2 tests or independent t-tests, as appropriate

### Regression analysis

The results of multiple linear regressions are provided in Table 2 (findings for the covariates are shown in Supplementary File 2). R² value was 0.15. The mean VIF was 2.20 and all VIFs were well below 10 suggesting that there are no multicollinearity problems. In our regression model, it was adjusted for gender, age, education, marital status, labor force participation, BMI, smoking status, alcohol intake, sports activities, physical functioning, self-rated health and the number of chronic conditions. Our regression model showed that sexual minorities reported a lower expected longevity (β=-0.69, p = .02) compared to heterosexuals. The partial eta-squared value for sexual minority was 0.06%. In a robustness check, we used a FIML approach to deal with missing data. The association between sexual minorities and a lower expected longevity remained very similar (β=-0.67, p = .02). In a further robustness check, the expected longevity was limited to 120 years. In this model, the association of interest also remained nearly identical (β=-0.63, p = .03). Moreover, we again used a FIML approach and limited the expected longevity to 120 years. Once more, the association of interest remained very similar (β=-0.61, p = .03). When our main model was extended by adding depressive symptoms as covariate, the association between sexual minorities and lower expected longevity remained similar (β=-0.61, p = .04 with listwise deletion; β=-0.59, p = .04).

**Table 2:** Sexual orientation and expected longevity. Results of multiple linear regressions (wave 5)

Independent variables	Expectations of longevity – with listwise deletion	Expectations of longevity – with FIML	Expectations of longevity – restricted to 120 years and with listwise deletion	Expectations of longevity – restricted to 120 years and with FIML
Covariates^†^	✓	✓	✓	✓
Sexual minorities (compared to heterosexuals)	-0.69* (0.29; -1.26 to -0.11)	-0.67* (0.29; -1.24 to -0.09)	-0.63* (0.29; -1.21 to -0.05)	-0.61* (0.29; -1.18 to -0.04)
Observations	6,424	7,698	6,419	7,690
R²	0.15	0.16	0.16	0.17

Comments: Unstandardized beta coefficients are displayed. Robust standard errors and 95% CI in parentheses. * p < .05. ^†^ Covariates include: gender, age, education, marital status, labor force participation, BMI, smoking status, alcohol intake, sports activities, physical functioning, self-rated health and the number of chronic conditions

We distinguished between all four categories of sexual orientation in a further robustness check. In this regression model, the differences in expected longevity were not significant when comparing heterosexuals and homosexuals (β=-0.43, p = .68). Moreover, marginal significant differences were identified between bisexual and heterosexual (β=-0.86, p = .09) as well as between other and heterosexual (β=-0.67, p = .06).

## Discussion

Drawing on a large, representative dataset, our purpose was to investigate the association between sexual orientation and perceived longevity among individuals aged 40 years and over. Regressions revealed that sexual minorities reported a lower expected longevity compared to heterosexuals. This association remained nearly constant in robustness checks. The effect size (partial eta-squared) was very small. However, the difference between heterosexuals and sexual minorities equals about eight months and thus exceeds, for example, the association between each additional chronic condition and perceived longevity (which is about five months for each additional chronic condition). Another example, when we focus on the association between migration background (comparison between: not having a migration background vs. having a migration background and experiencing migration) and perceived longevity, the association was only somewhat more pronounced (β = 1.00, p = .047) - compared to the association between sexual orientation and perceived longevity. To our best knowledge, as the first study we examined the association between sexual orientation and expected longevity. For this reason, our current findings are rather difficult to compare with prior research.

It may be the case that particularly individuals belonging to sexual minorities may fear stigma, rejection or discrimination in later life (e.g., in nursing homes [4, 8, 9]). For example, a former study [23] has also shown that individuals belonging to sexual minorities experienced discriminatory behavior in hospices. Therefore, due to the risk for receiving discriminatory end-of-life care and in line with the minority stress theory, individuals belonging to sexual minorities may feel that they die earlier compared to their heterosexual counterparts. We also found that lower life satisfaction (measured using the Satisfaction with Life Scale [24]; β = 0.97, p < .001) and more depressive symptoms (measured using the Center for Epidemiologic Studies Depression Scale, 15-item version [21]; β=-0.07, p < .01) were both associated with a lower perceived longevity.

Due to stressors mentioned above, individuals belonging to sexual minorities may also fear that their partner will die relatively young. As a consequence, they may fear that their partner cannot take care of her or him when in future need for care. This factors could also explain the association between sexual orientation and perceived longevity. Actually, when adding interaction terms (i.e., sexual minority x marital status), some of the interaction terms achieved statistical significance (e.g., sexual minority x married, living separated from spouse (compared to married, living together with spouse: β=-4.82, p = .01) – which may underline the role of marital status in the link between sexual orientation and perceived longevity.

We would like to highlight some strengths and limitations of our study. Overall, this is the very first study investigating the association between sexual orientation and expected longevity. Additionally, data were taken from a large, nationally representative sample. Furthermore, numerous covariates (including health-related factors) were included in regression analysis. Moreover, missing values were tackled using a FIML approach. However, some limitations are worth bearing in mind. Cross-sectional data were used with its known limitations regarding directionality. Comparable to other large cohort studies, single-items (with a high face validity) were used to quantify the variables of interest (i.e., sexual orientation and perceived life expectancy). In the DEAS study, a small selection bias has been identified [15]. While, among other things, several lifestyle-related as well as several health-related factors (physical functioning, self-rated health, number of chronic conditions and depressive symptoms) were included in our regression model, it may be the case that not all disease-related factors were accounted for – which could also explain the association between sexual minority and the perceived life expectancy.

In conclusion, beyond the impact of other covariates, an association between sexual minority and a lower perceived life expectancy exists. Efforts are required to make sexual minorities believe in a high life expectancy (e.g., increased optimism or reduced perceived discrimination) – which in turn can help to increase their actual longevity and successful ageing. Future research, e.g., based on qualitative designs, is required to explore underlying mechanisms (such as expected stigma in later life). Moreover, future research could focus on younger cohorts (see also: [25]). It could be that existing differences in perceived life expectancy according to sexual orientation disappear, e.g. because sexual minorities in such age cohorts feel less discriminated against.

### Electronic supplementary material

Below is the link to the electronic supplementary material.

Supplementary Material 1. Sample characteristics for the analytical sample (n = 6,424 individuals; 5,903 heterosexuals, 521 sexu-al minorities) – with column percentages

Supplementary Material 2. Sexual orientation and expected longevity. Results of multiple linear regressions (wave 5)

## Data Availability

The data used in this study are third-party data. The anonymized data sets of the DEAS (1996, 2002, 2008, 2011, 2014, 2017, 2020, 2020/2021) are available for secondary analysis. The data has been made available to scientists at universities and research institutes exclusively for scientific purposes. The use of data is subject to written data protection agreements. Microdata of the German Ageing Survey (DEAS) is available free of charge to scientific researchers for non-profitable purposes. The FDZ-DZA provides access and support to scholars interested in using DEAS for their research. However, for reasons of data protection, signing a data distribution contract is required before data can be obtained. Please see for further information (data distribution contract): https://www.dza.de/en/research/fdz/access-to-data/formular-deas-en-english. DEAS-Support is provided by Dr. Stefan Stuth (stefan.stuth@dza.de). Please see for further details: https://www.dza.de/en/research/fdz/contact-and-support.

## List of abbreviations

BMI: Body Mass Index
DEAS: German Ageing Survey
FIML: Full–Information Maximum Likelihood
ISCED: International Standard Classification of Education
VIF: Variance Inflation Factor
