# Supplementary material for: Do sexual minorities believe that they die earlier? Results from a large, representative survey
Source: BMC Geriatr. 2023 Nov 14;23:742. doi: 10.1186/s12877-023-04453-5 (PMC10648699; doi:10.1186/s12877-023-04453-5)
Supplement: Supplementary file 1 — Supplementary Material 1. Sample characteristics for the analytical sample (n = 6,424 individuals; 5,903 heterosexuals, 521 sexu-al minorities) – with column percentages [file 12877_2023_4453_MOESM1_ESM.docx]

Supplementary File 1. Sample characteristics for the analytical sample (n=6,424 individuals; 5,903 heterosexuals, 521 sexual minorities) – with column percentages

| Variables | Heterosexuals | Sexual  minorities | Total | P-value |
| --- | --- | --- | --- | --- |
| Expected longevity: Mean (SD) | 83.9 (7.7) | 83.5 (7.1) | 83.9 (7.7) | .22 |
| Gender: N (%) |  |  |  | .33 |
| Men | 2985 (50.6) | 275 (52.8) | 3260 (50.7) |  |
| Women | 2918 (49.4) | 246 (47.2) | 3164 (49.3) |  |
| Age: Mean (SD) | 63.2 (10.9) | 67.6 (11.0) | 63.6 (11.0) | <.001 |
| Education (ISCED-97): N (%) |  |  |  | <.001 |
| Low education (ISCED: 0-2) | 267 (4.5) | 49 (9.4) | 316 (4.9) |  |
| Medium education (ISCED: 3-4) | 2940 (49.8) | 297 (57.0) | 3237 (50.4) |  |
| High education (ISCED: 5-6) | 2696 (45.7) | 175 (33.6) | 2871 (44.7) |  |
| Marital status: N (%) |  |  |  | <.001 |
| Married, living together with spouse | 4221 (71.5) | 312 (59.9) | 4533 (70.6) |  |
| Married, living separated from spouse | 102 (1.7) | 5 (1.0) | 107 (1.7) |  |
| Divorced | 614 (10.4) | 56 (10.7) | 670 (10.4) |  |
| Widowed | 560 (9.5) | 84 (16.1) | 644 (10.0) |  |
| Single | 406 (6.9) | 64 (12.3) | 470 (7.3) |  |
| Employment status: N (%) |  |  |  | <.001 |
| Working | 2407 (40.8) | 126 (24.2) | 2533 (39.4) |  |
| Retired | 2956 (50.1) | 361 (69.3) | 3317 (51.6) |  |
| Other (not employed) | 540 (9.1) | 34 (6.5) | 574 (8.9) |  |
| Body-Mass-Index (BMI): Mean (SD) | 26.9 (4.6) | 27.3 (4.6) | 26.9 (4.6) | .06 |
| Smoking status: N (%) |  |  |  | <.01 |
| Yes, daily | 863 (14.6) | 61 (11.7) | 924 (14.4) |  |
| Yes, sometimes | 240 (4.1) | 15 (2.9) | 255 (4.0) |  |
| No, not anymore | 2251 (38.1) | 178 (34.2) | 2429 (37.8) |  |
| Never smoking | 2549 (43.2) | 267 (51.2) | 2816 (43.8) |  |
| Alcohol intake: N (%) |  |  |  | <.01 |
| Daily | 743 (12.6) | 83 (15.9) | 826 (12.9) |  |
| Several times a week | 1521 (25.8) | 107 (20.5) | 1628 (25.3) |  |
| Once a week | 972 (16.5) | 74 (14.2) | 1046 (16.3) |  |
| 1-3 times a month | 728 (12.3) | 58 (11.1) | 786 (12.2) |  |
| Less often | 1341 (22.7) | 130 (25.0) | 1471 (22.9) |  |
| Never | 598 (10.1) | 69 (13.2) | 667 (10.4) |  |
| Doing sport: N (%) |  |  |  | <.001 |
| Daily | 487 (8.3) | 40 (7.7) | 527 (8.2) |  |
| Several times a week | 1723 (29.2) | 113 (21.7) | 1836 (28.6) |  |
| Once a week | 1098 (18.6) | 80 (15.4) | 1178 (18.3) |  |
| 1-3 times a month | 455 (7.7) | 35 (6.7) | 490 (7.6) |  |
| Less often | 711 (12.0) | 59 (11.3) | 770 (12.0) |  |
| Never | 1429 (24.2) | 194 (37.2) | 1623 (25.3) |  |
| Physical functioning (from 0 (worst) to 100 (best): Mean (SD) | 83.3 (21.8) | 77.1 (25.2) | 82.8 (22.1) | <.001 |
| Self-rated health (from 1 = very good to 5 = very bad): Mean (SD) | 2.5 (0.8) | 2.7 (0.8) | 2.5 (0.8) | <.001 |
| Number of physical illnesses (count: 0 to 11 physical illnesses): Mean (SD) | 2.5 (1.8) | 3.0 (2.0) | 2.5 (1.8) | <.001 |
| Depressive symptoms (from 0 to 45, with higher values reflect more depressive symptoms): Mean (SD) | 6.4 (5.8) | 8.0 (6.6) | 6.5 (5.9) | <.001 |

Notes: P-values are based on χ2 tests or independent t-tests, as appropriate.
