# Supplementary material for: Do sexual minorities believe that they die earlier? Results from a large, representative survey
Source: BMC Geriatr. 2023 Nov 14;23:742. doi: 10.1186/s12877-023-04453-5 (PMC10648699; doi:10.1186/s12877-023-04453-5)
Supplement: Supplementary file 2 — Supplementary Material 2. Sexual orientation and expected longevity. Results of multiple linear regressions (wave 5) [file 12877_2023_4453_MOESM2_ESM.docx]

Supplementary File 2. Sexual orientation and expected longevity. Results of multiple linear regressions (wave 5)

| Independent variables | Expectations of longevity – with listwise deletion | Expectations of longevity – with FIML | Expectations of longevity – restricted to 120 years and with listwise deletion | Expectations of longevity – restricted to 120 years and with FIML |
| --- | --- | --- | --- | --- |
|  |  |  |  |  |
| Sexual minorities (Ref.: Heterosexuals) | -0.69* | -0.67* | -0.63* | -0.61* |
|  | (0.29) | (0.29) | (0.29) | (0.29) |
| Gender: Women (Ref.: Men) | -0.17 | 0.27*** | -0.14 | 0.28*** |
|  | (0.21) | (0.02) | (0.19) | (0.01) |
| Age (in years) | 0.25*** | 0.00 | 0.26*** | 0.00 |
|  | (0.02) | (0.01) | (0.01) | (0.00) |
| Education (ISCED-97): - Medium (Ref.: Low) | 0.20 | -1.69*** | 0.15 | -1.59*** |
|  | (0.42) | (0.15) | (0.42) | (0.14) |
| - High | 0.55 | -0.43*** | 0.41 | -0.43*** |
|  | (0.44) | (0.06) | (0.43) | (0.05) |
| Marital status: - Married, living separated from spouse (Ref.: Married, living together with spouse) | 0.83 | -0.00 | 0.90 | -0.03 |
|  | (0.68) | (0.03) | (0.68) | (0.02) |
| - Divorced | -0.04 | -0.22 | -0.14 | -0.20 |
|  | (0.34) | (0.20) | (0.30) | (0.19) |
| - Widowed | 0.41 | 0.08 | 0.42 | 0.02 |
|  | (0.28) | (0.35) | (0.27) | (0.35) |
| - Single | 0.34 | 0.47 | 0.43 | 0.36 |
|  | (0.38) | (0.37) | (0.37) | (0.37) |
| Employment status: - Retired (Ref.: Employed) | -1.45*** | 0.71 | -1.71*** | 0.77 |
|  | (0.35) | (0.65) | (0.31) | (0.66) |
| - Other (not employed) | -1.56*** | 0.02 | -1.80*** | -0.08 |
|  | (0.42) | (0.33) | (0.37) | (0.30) |
| Physical functioning (SF-36 subscale; from 0 (worst) to 100 (best)) | 0.00 | 0.39 | 0.00 | 0.42+ |
|  | (0.01) | (0.25) | (0.01) | (0.25) |
| Self-rated health (from 1 = very good to 5 = very bad) | -1.75*** | 0.27 | -1.65*** | 0.36 |
|  | (0.16) | (0.36) | (0.14) | (0.36) |
| Number of physical illnesses | -0.43*** | -1.66*** | -0.44*** | -1.88*** |
|  | (0.06) | (0.33) | (0.06) | (0.30) |
| Body-Mass-Index (kg/m²) | 0.00 | -1.63*** | -0.02 | -1.84*** |
|  | (0.03) | (0.40) | (0.02) | (0.35) |
| Smoking: - Yes, occassionally (Ref.: Yes, daily) | 2.01*** | 1.93*** | 2.13*** | 2.04*** |
|  | (0.56) | (0.54) | (0.55) | (0.53) |
| - No, not anymore | 1.68*** | 1.63*** | 1.74*** | 1.66*** |
|  | (0.31) | (0.30) | (0.30) | (0.29) |
| - Never smoking | 1.75*** | 1.66*** | 1.76*** | 1.66*** |
|  | (0.31) | (0.30) | (0.30) | (0.29) |
| Alcohol intake: - Several times a week (Ref.: daily) | 0.08 | 0.08 | 0.05 | 0.13 |
|  | (0.30) | (0.30) | (0.28) | (0.27) |
| - Once a week | 0.23 | 0.20 | 0.26 | 0.29 |
|  | (0.33) | (0.33) | (0.32) | (0.31) |
| - 1-3 times a month | 0.16 | 0.12 | 0.25 | 0.27 |
|  | (0.35) | (0.34) | (0.34) | (0.33) |
| - Less often | 0.36 | 0.33 | 0.38 | 0.43 |
|  | (0.32) | (0.31) | (0.30) | (0.29) |
| - Never | -0.02 | -0.00 | 0.09 | 0.16 |
|  | (0.39) | (0.37) | (0.38) | (0.36) |
| Frequency of sports activities: - Several times a week (Ref.: daily) | 0.05 | 0.16 | 0.08 | 0.19 |
|  | (0.32) | (0.31) | (0.32) | (0.31) |
| - Once a week | -0.40 | -0.35 | -0.44 | -0.38 |
|  | (0.34) | (0.32) | (0.34) | (0.32) |
| - 1-3 times a month | 0.19 | 0.39 | 0.00 | 0.21 |
|  | (0.47) | (0.45) | (0.43) | (0.41) |
| - Less often | -0.62 | -0.53 | -0.57 | -0.49 |
|  | (0.38) | (0.37) | (0.38) | (0.37) |
| - Never | -0.28 | -0.28 | -0.40 | -0.42 |
|  | (0.35) | (0.32) | (0.34) | (0.31) |
| Constant | 72.47*** | 71.42*** | 72.16*** | 71.19*** |
|  | (1.65) | (1.46) | (1.49) | (1.35) |
|  |  |  |  |  |
| Observations | 6,424 | 7,698 | 6,419 | 7,690 |
| R² | 0.15 | 0.16 | 0.17 | 0.17 |

Comments: Unstandardized beta coefficients are displayed. Robust standard errors in parentheses; *** p<0.001, ** p<0.01, * p<0.05, + p<0.10
